# ﻿Taxonomic revision of the *Cyathulaachyranthoides* group (Amaranthaceae, Achyranthoids) in continental Africa and Madagascar

**DOI:** 10.3897/phytokeys.260.162975

**Published:** 2025-08-01

**Authors:** Alexander P. Sukhorukov, Alexander N. Sennikov, Martin Cheek, Louis Nusbaumer, Maria Kushunina, Alexander C. Timonin

**Affiliations:** 1 Department of Higher Plants, Biological Faculty, Lomonosov Moscow State University, 119234, Moscow, Russia; 2 Botanical Museum, Finnish Museum of Natural History, P.O. Box 7, 00014 University of Helsinki, Helsinki, Finland; 3 Royal Botanic Gardens, Kew, Richmond, Surrey, TW9 3AE, UK; 4 Conservatoire et Jardin botaniques de Genève and Plant Biodiversity Centre, Department of Plant Sciences, University of Geneva, ch. de l’Impératrice 1, C.P. 71, 1292 Chambésy, Switzerland; 5 Department of Plant Physiology, Biological Faculty, Lomonosov Moscow State University, 119234, Moscow, Russia

**Keywords:** Africa, *Cyathula*, distribution, florescence, Madagascar, morphology, taxonomy

## Abstract

*Desmochaeta* (now *Cyathula*) *achyranthoides* was described from South America and reported to be a widespread tropical plant in both Africa and the Americas. A revision of herbarium material revealed that *inter alia* leaf shape differs between the populations of the Eastern and Western Hemispheres. Therefore, we maintain the name *C.achyranthoides* s.str. for the American populations and re-instate the name *C.geminata* for most of the African plants. Both species are found in tropical evergreen forests, mainly at low altitudes. Furthermore, two mountain species, *C.brevispicata* from Madagascar and *C.aethiopica* from east tropical Africa, which were previously identified as *C.achyranthoides*, are described as new to science. Compared to both *C.achyranthoides* and *C.geminata*, these new species have short inflorescences and longer, recurved or uncinate perianths in the fertile flowers and morphologically resemble *C.fernando-poensis*; the latter is only known from the mountains of Equatorial Guinea (Bioko Island), south-west and (newly recorded here) North-West Regions of Cameroon. The species under study are compared with one another and with the related, pantropically distributed species *C.prostrata*; their synonymy is verified and typifications are established. The fine-level partial florescence (cyme) structure of each species is also studied, with further taxonomic implications. *Cyathulageminata* seems to be restricted to west and central tropical Africa, with its range replaced eastwards by *C.aethiopica* and in Madagascar by *C.brevispicata*.

## ﻿Introduction

The taxonomic composition of *Cyathula* has drastically changed after the phylogenetic revision of the entire achyranthoid clade and the genus now comprises ~ 40 species distributed in the Tropics (Di Vincenzo et al. 2025). Under its revised circumscription, *Cyathula* incorporates the previously recognised genus *Nelsia* Schinz and part of *Sericocomopsis* Schinz and is now considered the largest genus within the achyranthoid clade. *Cyathula* includes plants with various life forms, inflorescence and flower structure, but in the vast majority of cases, the partial florescences (cymes) have both fertile and sterile flowers (Acosta et al. 2009). Perianth and bracteoles of the sterile flowers can be hooked at the tip, as is the case in two widespread tropical species, *C.prostrata* (L.) Blume (the type of the genus) and *C.achyranthoides* (Kunth) Moq. These species are phylogenetically related and represent sister lineages, based on combined plastid DNA regions or form a subclade together with some other achyranthoids (Di Vincenzo et al. 2025).

*Cyathulaprostrata* and *C.achyranthoides* share several important morphological characters, including a rooting habit, short-petiolate leaves that are rhombic, elliptic or obovate and cymes with an uncinate sterile part that facilitates epizoochory. They differ in the number of fertile flowers in a cyme (one or two in *C.achyranthoides* vs. three in *C.prostrata* (e.g. Hutchinson and Dalziel (1927); Hauman (1951); Duke (1961); Cavaco (1962)) and in the length of the perianths of the fertile flowers (3.0–5.0 mm vs. 2.0–3.0 mm long (e.g. Standley (1937b); Backer (1948); Cavaco (1962); Townsend (1985)).

*Cyathulaprostrata* is a common plant in the Tropics, found in primary forests as well as in anthropogenically degraded semi-shady communities, for example, in orchards and along trails (e.g. Hutchinson and Dalziel (1927); Backer (1948); Townsend (1980)). It seems to be native to the Old World and is considered introduced in Polynesia (Florence 2004) and in the Americas (Standley 1937b; Duke 1961; Borsch 2001; Agudelo Henao 2008a, b; Acevedo-Rodríguez and Strong 2012). Nevertheless, the alien status of the species in the Americas has not been fully clarified. On the one hand, *C.prostrata* was reported for the flora of Trinidad and Tobago (Moquin-Tandon 1849), based on a specimen probably collected by F. von Wrbna in 1822 and later distributed by F.W. Sieber (see also the specimen P00609948). On the other hand, *C.prostrata* was not mentioned in the later floristic accounts of some American countries, for example, in Central America (Hemsley 1882; Urban 1909; Standley and Record 1936; Greuter and Rodríguez 2017), Surinam (Pulle 1906) and Peru (Standley 1937a), but is now widely distributed across the Tropics of South and Central America (GBIF Secretariat 2023).

*Cyathulaachyranthoides* is said to be distributed across tropical Africa and America and is supposed to be alien in the New World (Borsch 2001; Funk et al. 2007). The alien status of the species in the Americas has not yet been confirmed by detailed study, but the species was originally described from Colombia (Kunth (1818), as *Desmochaetaachyranthoides* Kunth and *D.densiflora* Kunth) and was also known in the early 19^th^ century from other American countries as can be seen from von Martius (1826a, 1826b, as *Pupaliadensiflora* (Kunth) Mart.) and an early herbarium specimen collected in Saint Vincent and the Grenadines (K005773490!). Schinz (1893) reported *C.achyranthoides* both from Africa and South America.

Notably, some details in the morphological descriptions from different treatments lack compatibility, especially for the African populations. For instance, the inflorescence is 3–15 cm long in the East and West African plants (Cavaco 1962; Townsend 1985), which generally corresponds with the measurements in the American specimens (Standley 1917; Senna et al. 2010), but in the Madagascan plants, the inflorescence is only 1–4 cm long (Cavaco 1953, 1954). Besides, Townsend (1985) indicated a variable (3–5 mm) length of the fertile flower in a cyme. We have also noted some other discrepancies in *C.achyranthoides* s.l. across tropical Africa, for example, different florescence structure, variable pubescence of the perianths and different altitude preferences. As a result, there is no doubt that the species is not morphologically uniform in the Old World. Concomitantly, there is a striking difference between the African and American plants in leaf shape. These facts encouraged us to undertake a revision of *Cyathulaachyranthoides* in Africa, with further comments on the distributions and geographical patterns of this aggregate.

## ﻿Materials and methods

Herbarium specimens were examined and (re)identified in BM, G, K, LE, MW and MHA. The virtual Herbaria of B, BR, F, FT, INB, L, MA, NY, P, RB, TOGO, U, US, USF and WAG were also used when the images allowed exact identification. One specimen from DR Congo was borrowed from BR with further examination in G.

Images of reproductive diaspores were photographed with a Nikon DS-Vi1 camera at the Department of Higher Plants (Moscow State University). Distribution maps were prepared using the SimpleMappr online tool (http://www.simplemappr.net).

We have studied the partial florescences (cymes) for better understanding their structure with further systematic implications. The structure of the inflorescences is interpreted and described according to Troll (1964) and Kuznetsova and Timonin (2017).

## ﻿Results

### ﻿Taxonomic part

The American and African populations of *Cyathulaachyranthoides* differ in leaf shape and, based on that, we accept the name *C.geminata* for most of the African plants. Both species are tropical lowland or submontane plants with pubescent perianths. In our opinion, the morphological descriptions of both species should be modified. Three other species growing at higher elevations and having larger (sub)glabrous perianths and other distinct characters deserve their own specific status and two of them are described here as new to science.

We have paid special attention to the inflorescence structure of the species under consideration, because the five species accepted in the *C.achyranthoides* group have two different types of the inflorescence architecture.

### ﻿Key to the species of the *Cyathulaachyranthoides* group

**Table:** 

1	Cymes visually asymmetric (with a sterile part and a fertile flower); perianth of fertile flowers 3.5–4.0 mm long, pubescent, but often glabrescent at fruiting, its segments straight at the apex (neither uncinate nor deflexed)	**2**
–	Cymes symmetric (two sterile parts surrounding a fertile flower), but occasionally asymmetric in some cymes; perianth of fertile flowers 4.5–6.0 mm long, glabrous or hairy at the base, apically uncinate or deflexed, rarely straight	**3**
2	Leaves oblong; inflorescence 70–150(170–200) mm long. Tropical America	***C.achyranthoides***
–	Leaves rhombic, ovate or obovate; inflorescence up to 100 mm long. Tropical Africa	***C.geminata***
3(1)	Inflorescence up to 25(30) mm long; style 1.2–1.6 mm long. Madagascar	***C.brevispicata***
–	Inflorescence up to 60 mm long; style not exceeding 1.1 mm. Plants from other parts of Africa	**4**
4	Perianth segments of fertile flowers straight or slightly deflexed at the apex, ± glabrous. West Africa (Bioko Island and SW and NW Regions of Cameroon)	***C.fernando-poensis***
–	Perianth segments of fertile flowers usually hairy at the base, uncinate at the apex, or both uncinate and mucronulate perianths present in the same plant. East Africa	***C.aethiopica***

### ﻿Taxonomic treatment

#### ﻿ Cyathula achyranthoides

Taxon classificationPlantaeCaryophyllalesAmaranthaceae

(Kunth) Moq. in DC., Prodr. 13(2): 326 (1849)

0747A6D3-C787-53E1-8565-7B483AA6560F

Fig. 1

≡ Desmochaetaachyranthoides Kunth, Nov. Gen. Sp. 2: 210 (1818).
≡ Cyathulaprostratavar.achyranthoides (Kunth) Kuntze, Revis. Gen. Pl. 2: 542 (1891).
= Desmochaetadensiflora Kunth, Nov. Gen. Sp. 2: 211 (1818).
≡ Pupaliadensiflora (Kunth) Mart., Nov. Gen. Sp. Pl. 2(1): 61, t. 156, 158 (1826).
≡ Cyathulaachyranthoidesvar.densiflora (Kunth) Moq. in DC., Prodr. 13(2): 327 (1849). Holotype: [Colombia, Bolívar Department?] Rio Magdalena, [1801] Herb. Humboldt & Bonpland (P00670029 – image seen!; isotype P00136028 – image seen!). Note. Kunth (1818) stated that the collections of both species, Desmochaetaachyranthoides and D.densiflora, originated from the same locality at Mompox. However, the original label of the type collection of the latter species does not mention Mompox, but only the Rio Magdalena River. This unnumbered collection could have originated elsewhere along the river, like at Bojorque [Bohorquez] as was previously stated on the type label. Kunth (1818) noted the differences between Desmochaetaachyranthoides and D.densiflora as follows: stems pubescent vs. glabrous, leaves longer vs. shorter, acuminate vs. acute, pubescent vs. less hairy, inflorescences slightly vs. densely pubescent, perianth [of the fertile flowers] throughout finely vs. basally and apically pubescent. These differences seem to be insignificant and likely fall within the natural variability of C.achyranthoides. It should be noted that the perianth segments lose their pubescence during fruiting, which often makes them appear more glabrous than they do at flowering.
= Desmochaetauncinata Willd. ex Roem. & Schult., Syst. Veg., ed. 15, 5: 554 (1819). Holotype: [Colombia, Bolívar Department, Santa Cruz de Mompox, Rio Magdalena, April 1801], A. Humboldt 1507 (B-W05006-020 – image seen!; isotypes P00670028, P00136029). Note. As follows from the collection number and plant characters, this species was described on the basis of a duplicate specimen from the type collection of Desmochaetaachyranthoides Kunth. Due to the brevity of the original description and the lack of the collection number in the protologue of Desmochaetauncinata Willd. ex Roem. & Schult., Kunth (1820) was not able to recognise the synonymy when he aligned the South American taxa described from the collection of Willdenow with his own species.
= Cyathulaachyranthoidesvar.glabrescens Moq. in DC., Prodr. 13(2): 327 (1849). Holotype: Guiane anglaise [Guyana], 1839, Schomburgk 697 (G-DC [G00688954!]; isotype P00609947 – image seen!). Note. Moquin-Tandon (1849) indicated that this variety was based on a single specimen at G-DC, which is the holotype. That specimen represents a stem collected in fruit. The hairs on the fertile flowers have partially fallen off; for this reason, the plant looks less hairy.
= Achyrantheshirtiflora A.Rich. in Sagra, Hist. Fis. Cuba 11: 175 (1850). Lectotype (designated here). Cuba, Ramon de la Sagra s.n. (P04558206–image seen!).

##### Holotype.

[Colombia, Bolívar Department] Rio Magdalena, [Santa Cruz de] Mompox, [April 1801], Herb. Humboldt & Bonpland 1507 (P00670028 – image seen!; isotypes B-W05006-020 – image seen!, P00136029 – image seen!). Note. The type specimens have been curatorially mislabelled as collected at Bojorque [Bohorquez], which is situated in the Atlántico Department of Colombia.

**Figure 1.**
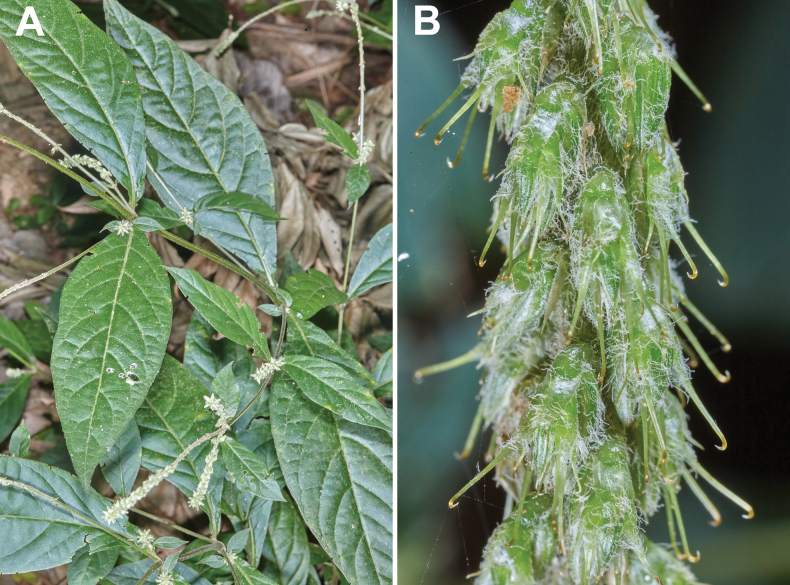
*Cyathulaachyranthoides*. A. Leaves; B. Inflorescence (Nicaragua, El Brujo, 11.97984, -86.26455). Photographer: Sune Holt.

##### Note.

This species was cited as a synonym of *Cyathulaachyranthoides* (Standley 1917; Duke 1961; Acevedo-Rodríguez and Strong 2012; Greuter and Rodríguez 2017) or *C.prostrata* (Grisebach 1866; Sauvalle 1870). In fact, one of the two specimens available, P04558205, represents a true *Achyranthes* with glabrous flowers arranged in spikes. This specimen is *A.fruticosa* Lam., which is native to Central America (Sukhorukov et al. 2024). The other specimen, P04558206, which is selected here as lectotype is *Cyathulaachyranthoides*. This choice is in agreement with the original description of *Achyrantheshirtiflora* whose flowers were described as lanate (Richard 1850).

##### Description.

**Figure 2.**
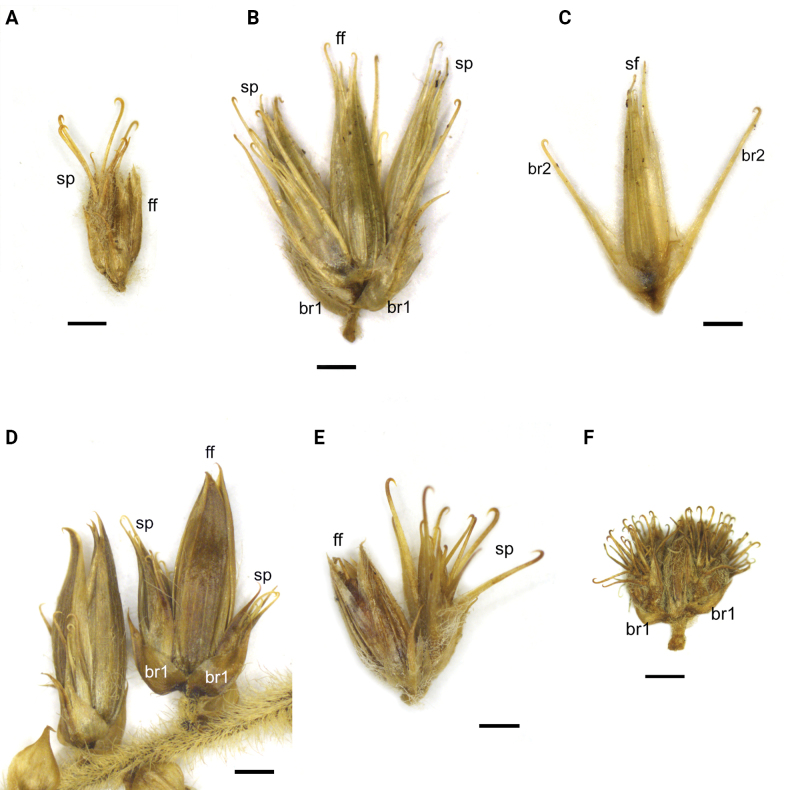
Cymes of studied *Cyathula* species. A. *C.achyranthoides*; B. *C.brevispicata*; C. Sterile flower of *C.brevispicata* with bracteoles; D. *C.fernando-poensis*; E. *C.geminata*; F. *C.prostrata*. Abbreviations: br1, br 2 – bracteoles of the first and second orders, sp – sterile part, ff – fertile flower. Scale bars: 1 mm.

**Figure 3.**
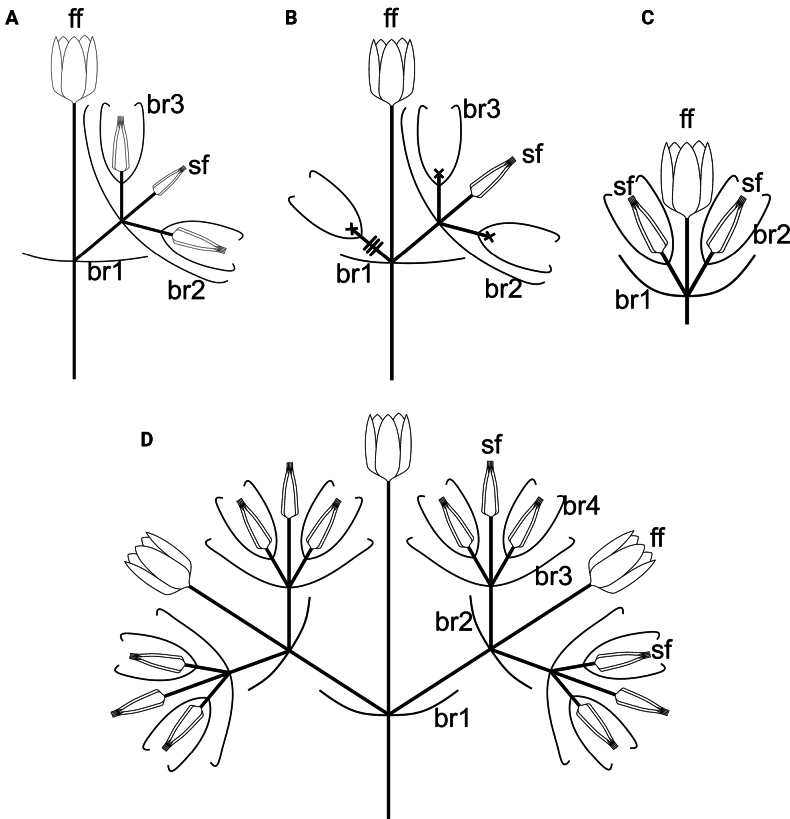
Simplified cyme architecture in the *Cyathulaachyranthoides* group and related *C.prostrata*. A, B. Asymmetric cymes of *C.achyranthoides* and *C.geminata* including the common type (A) with a sterile part with one sterile flower (sf) located on one side of the fertile flower (ff), and deviating type (B) with one sessile rudimentary flower or bracteole(s); C. Symmetric cyme of *C.aethiopica*, *C.brevispicata* and *C.fernando-poensis* with one fertile flower (ff) and two sterile flowers (sf) located laterally; D. Symmetric cyme of *C.prostrata* with three fertile flowers and sterile parts with sterile flowers. Abbreviations: *br1*, *br2*, *br3*, and *br4* – uncinate or non-uncinate bracteoles of different orders, X – rudimentary flowers of the sterile part.

***Perennial herbs*** up to 1 m high, rooting at nodes (sometimes flowering in the first year and resembling an annual); ***leaf pairs*** 3–4 on each branch; ***leaves*** oblong, shortly petiolate (petioles up to 15 mm long), apically long-attenuate, 40–150(170) × 20–50(70) mm, sparsely pubescent below with appressed hairs mostly on the veins, sometimes glabrous on both sides; ***bract*** subtending each cyme (persistent on the inflorescence axis) 2.0 mm long; ***synflorescences*** rather dense or rarely interrupted in lower part, main florescence 70–150(170–200) mm long, paracladia present; ***cymes*** (Fig. 2A) asymmetric, with two unequal, narrowly oblong, mucronate first-ordered bracteoles (or *br1*: Fig. 3A, B) and consisting of ***fertile part*** of a cyme (one perfect flower) and ***sterile part*** mostly located on one side of the fertile flower (sometimes one very short and almost unnoticeable rudimentary flower is located on the other side of the fertile flower); ***sterile part*** with a pedicel 1.0–1.5 mm long, bearing one sterile flower with perianth segments and two uncinate second-ordered bracteoles (*br2*) 2.5–4.0 mm long, each *br2* has axillary highly rudimentary flowers accompanied by paired bracteoles beneath (*br3*) shorter than *br2*; ***sterile flower*** with a narrowly cylindrical perianth 1.5–2.5 mm long, acuminate or rarely two of five segments ± uncinate; thus, each cyme has at least 6(8) unequal uncinae; ***fertile flower*** with five green perianth segments 3.0–4.0 mm long, pubescent with curved hairs and often glabrescent at fruiting, each segment mucronulate and three-nerved; ***pseudostaminodes*** 0.2–0.3 mm long, entire; ***anthers*** 0.3–0.4 mm long; ***style*** (with capitate stigma) 0.6–1.0 mm long; ***fruit*** (without style) 1.8–2.4 mm long; ***seed coat*** brown, thin; ***radicle*** pointing upwards.

##### Habitat.

Tropical forests up to 1150(1500) m a.s.l. and rough ground; a common weed in many localities of Central America (Standley and Steyermark 1946; Burger 1983). The presence at higher elevations in Colombia (1800 m a.s.l.: Schellenberg et al. (1914)) is either exceptional or erroneous. In the West Indies, the species is mostly restricted to the Greater Antilles (Acevedo-Rodríguez and Strong 2012) and absent or very rare on the Lesser Antilles (Kellogg 1988).

##### IUCN Category.

Besides the natural habitat of *C.achyranthoides* in forests, the species is also found on rough ground, being a noxious weed in many countries (see above). We evaluate *C.achyranthoides* as a Least Concern (LC) species.

##### Distribution

**Figure 4.**
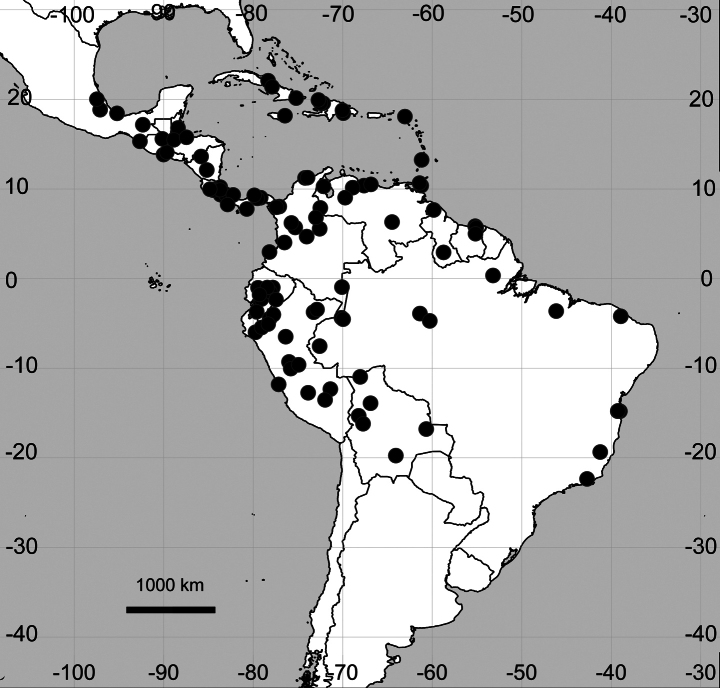
Distribution map of *Cyathulaachyranthoides*.

**(Fig. 4). Belize**: [Stann Creek Distr.] All Pines, 11 Mar 1932, W.A. Schipp 777 (K005773478).

**Bolivia**: [La Paz Dept.] Mapiri, 5000 ft [1524 m], Apr 1886, H.H. Rusby 1512 (K005773564); Yungas, 1890, M. Bang 505 (K005773563); Polo-Polo nr Coroico, 1912, O. Buchtien 686 (G, K005773562); Cochabamba Dept., San Rafaél, 24 Nov 1966, R.F. Steinbach 531 (F1645273, U1049813); Pando Dept., Nicolás Suárez, 15 Jan 1983, J.F. Casas 257610 (G); Beni Dept., José Ballivián Prov., 200 m a.s.l., 14 Dec 1994, J. Balderrama 373 (B100720368).

**Brazil**: nr Rio de Janeiro, Nov 1879, M. Glaziou (K); Rio Jurua, Oct 1900, E. Ule 5230 (G, K00111421); [Ceará State] Serra de Baturité, Sep 1910, E. Ule 9027 (G, K001207192, L1690980, NY00837888); Amazonas State, Rio Branco, 1912, J.G. Kuhlmann 2851 (RB00038465); Espirito Santo State. Rio Doce, Sep 1950, J.N. Vieira 12 (RB00038438); Amazonas State, Benjamin Constant Mun., 7 Sep 1962, A.P. Duarte 6856 (RB00038470); Bahia State, Ilheus, 2 Apr 1965, R.P. Belém & M. Magalhaes 634 (K001207199, NY00493802); [Acre State] Rio Jurua, nr Cruzeiro do Sul, 27 Oct 1966, G.T. Prance & al. 2907 (F1734964, K001207198, NY01212749, U1049762); Amazonas State, Beruri, 7 Apr 1967, M. Silva 810 (NY01212751); Paraná do Autáz-Mirim, 26 Aug 1973, C.C. Berg & al. 19768 (K001207201, NY01212750, U1049754); Bahia State, Itabuna, 30 m a.s.l., 9 Mar 1977, R.M. Harley 19482 (K001207200, NY00493799, P05003751, U1049753); Mun. Ilheus, area de CEPEC (Centro de Pesquisas do Cacau), 22 km from Rodovia, 4 Feb 1982, J.L. Hage 1627 (K001207202); Maranhão State, Caru Reserve, 17 Jul 1996, L. Cormier 164 (NO0052749).

**Colombia (selected)**: [Magdalena Dept.] Santa Marta, 1898–1901, H.H. Smith 1203 (G, K005773537); [Chocó Dept.] nr La Esmeralda, 750 m a.s.l., 19 Mar 1927, E. P. Killip & A.C. Smith 20925 (US03543074); Boyacá Dept., Labranzagrande, 1150 m a.s.l., 1931, D.G. Amórtegui 339 (US03543072); Bogotá Region, Mt. Chapon, 3500 ft, 7 Jun 1932, A.E. Lawrance 171 (G, K005773538); Cauca Dept., between Gorgona & Puerto Cabuyo, 1000 m a.s.l., 4 Jun 1943, J. Cuatrecasas 14503 (G); Antioquia Dept., Sonsón Mun., 23 Jan 1947, G. Gutiérrez 35544 (F1316964); [Antioquia Dept.] Heliconia, 28 Jun 1947, H. Daniel 3977 (F1283400); Rio Apaporis, between Rio Pacoa & Rio Kananari, 17 Jun 1951, R.E. Schultes & I. Cabrera 12605 (K005773539, US03543061); Rio Cacuetá, 2 May 1952, R.E. Schultes & I. Cabrera 16350 (K005773540); [Chocó Region] Calima Valley, 2500 ft [762 m], 5 Sep 1962, D. Hugh-Jones 386 (K005773541); Santander Dept., Humid canyon, 700 m a.s.l., 18 Jul 1965, F.A. Barkley 35205 (WAG1290508); Chocó Dept., W of Unguía, 17 Jul 1975, A. Gentry & L.E. Aguirre 15226 (F1841364); between Rios Vaupes & Apaporis, 17 Sep 1976, J.L. Zarucchi 2066 (K005773530).

**Costa Rica (selected)**: Guacimo, Aug 1901, anonymous 14669 (K00573477); San José Prov., nr El General, 950 m a.s.l., Nov 1936, A.F. Skutch 2904 (K005773482); Alajuela prov., Atenas, 28 Feb 1940, A. Smith 2467 (K005773483); Heredia Prov., Finca la Selva, 100 m a.s.l., 1 May 1981, J. Folsom 9978 (F2004755); Limón Prov., Talamanca, 3 Oct 2000, L. Acosta & al. 2820 (INB0003812629); Cartago Prov., Turrialba, 15 Jan 2001, E. Mora & E. Rojas 1756 (G); Puntarenas, 7 Nov 2006, L.D. Vargas & K. Rosales 1838 (INB0004204213).

**Cuba**: [Ciego de Ávila Mun.] La Cunagua, 19 Feb 1916, N.L. Britton & al. 14585 (NY01374422); Guantánamo, 26 Dec 1918, B. Huram 2280 (NY01374418); Cienfuegos Prov., Buenos Aires, Las Lagunas, 500–550 m a.s.l., 2 Feb 2023, E.R. Bécquer & al. 91773 (B101259197).

**Dominican Rep.**: Santo Domingo, 7 Feb 1929, E.L. Ekman 11477 (U1049885); [Monte Plata Prov.] Yamasá, 8 Apr 1978, Alain & Liogier 27503 (NY00493804).

**Ecuador (selected)**: [Canar Region] Southern Naranjapata, 550 m a.s.l., H.J.F. Schimpf 482 (G); Pastaza Prov., Tena, 3 Oct 1939, E. Asplund 9020 (K005773535); [El Oro Prov.] Zaruma, 1150 m a.s.l., 12 Aug 1947, R. Espinosa 1713 (F1727575); Los Ríos Prov., 65 m a.s.l., 26 Aug 1949, S.S. White 5615 (NO0052752); Napa-Pastaza Prov., Mera, 1100 m a.s.l., 20 Feb 1956, E. Asplund 19486 (G, K005773536); Napo Prov., Rio Napo, Chiroisla, 24 Aug 1979, L. Holm-Nielsen & al. 19781 (K005773544); Napo Prov., Rio Aguaroci, San Pablo de los Secoyas, 13 Feb 1980, L. Holm-Nielsen & al. 21074 (K005773502, U1049812); Morona-Santiago Prov., Taisha, 400 m a.s.l., 24 Jun 1980, J. Brandbyge & E. Asanza 32240 (K005773546); Pastaza Prov., Montalvo, 300 m a.s.l., 28 Jul 1980, B. Øllgaard & al. 35420 (F2019727, K005773545).

**El Salvador**: Ahuachapán Dept., San Francisco Menéndez, 380 m a.s.l., 13 Jan 2000, J.M. Rosales 32 (B100019089); Santa Ana Dept., Candelaria de la Frontera Mun., La Criba, 809 m a.s.l., 17 Apr 2013, D. Rodríguez & al. 4061 (B100043231).

**French Guiana**: Montagne Cacao, 6 July 2012, G. Léotard (https://www.gbif.org/occurrence/4978245017).

**Guatemala**: Alta Verapaz Dept., Cubilhuitz, 1901, H. von Türckheim 7966 & 11423 (G, US03543016); Izabal Dept., Los Amates to Izabal, 31 May 1919, S.F. Blake 7801 (US03543014).

**Guyana**: [without locality] 1841, Schomburgk 93 (G, K005773507); Barima-Waini Region, 5 miles [8 km] W of Arakaka, 20–80 m a.s.l., 7 Aug 1986, J. Pipoly & H. Lall 8348 (US00474940); Kanuku Mts., head of Nappi Creek, 31 Oct 1987, M.J. Jansen-Jacobs & al. 588 (K005773498).

**Haiti**: Dept. du Nord, 19 Nov 1924, E.L. Ekman 2574 (G, K005773493); nr Plaisance, 400 m a.s.l., 26 Jan 1926, E.C. Leonard 9198 (NY01374416); nr St. Louis du Nord, 1929, E.C. Leonard & G.M. Leonard 14360 (K005773486, NY01374413).

**Honduras**: Atlántida Dept., Tela, 18 Aug 1979, R. Andino 55 (US03542992); Atlántida Dept., Tela Mun., 10 Sep 2002, J. Pipoly & C. Valle 24307 (USF277322).

**Jamaica**: [without exact location] Mar 1871, ex herb. Hookerianum 1237 (K005773491); Port Antonio, 7 Jan 1906, A.E. Wight 36 (NY01374425); Bluefields, 500 m a.s.l., Mar 1908, N.L. Britton & A. Hollick 1999 (NY01374424).

**Mexico (selected)**: [Veracruz State] Orizaba, 1865–1866, M. Bourgeau 3040 (K005773473); Chiapas State, Escuintla, 22 Nov 1947, E. Matuda 17177 (K005773474); Veracruz State, San Andres Tuxtla, 450 m a.s.l., 12 Aug 1972, R. Cedillo 270 (BR0000027811986); Veracruz State, San Andres Tuxtla, 12 Aug 1974, R. Cedillo 270 (K005773476); State of Chiapas, Yajalón, 31 Aug 1983, A.M. Ton 6563 (MA614037); State of Puebla, Taxipehuatl, 2 Nov 2014, P. Acevedo-Rodríguez & al. 15965 (US01343465).

**Nicaragua**: Chontales [Dept.], 1867, R. Tate 334 (K005773472); Jinotega Dept., Wiwilí Mun., 165 m a.s.l., 10 Jun 2007, I. Coronado & A. Fernández 4019 (BM001172464).

**Panama (selected)**: [without exact locality] Nov 1861, S. Mayrs 612 (K005773533); nr Paya, 12 Jun 1959, W.L. Stern & al. 402a (G); Darién Prov., Rio Chico, 19 Dec 1966, D. Burch & al. 1083 (K005773552); Canal Zone, Farfan Beach, 27 Dec 1966, D. Burch & al. 1403 (K005773551); Bocas del Toro, Jun 1967, W.H. Lewis & al. 1997 (K005773549); Bocas del Toro, Chiriqui Prov., nr Puerto Armuelles airport, 17 Feb 1973, T.B. Croat 21885 (USF110618); Cocle & Herrera prov., border of Verauas, 11 Feb 1982, S. Knapp & al. 3338 (K005773553); nr Tiger Key, 21 Feb 1989, P.M. Person & C.R. Annable 6957 (K005773548).

**Peru (selected)**: [without exact location] 1832, Poppig 146 (G00688958); Huánuco Prov., 10 km S of Tingo Maria, 700 m a.s.l., 26 Oct 1938, H.E. Stork & O.B. Horton 9508 (F1078734, G, K005773560); Ganso Azul, 1500 ft [457 m], Oct 1942, Sandeman 3364 (K005773559); San Martín Region, nr Tarapoto, 890 m a.s.l., 11 Mar 1947, F. Woytkowski 35229 (F1316573, G); Bagua prov., E of Olmos, 22 Jan 1964, P.C. Hutchinson & J.K. Wright 3717 (F1640136, K005773561, P05002116); [Lambayeque Dept.] nr Olmos, 500 m a.s.l., 22 Jan 1964, P.C. Hutchinson & J.K. Wright 3717 (G); [Cuzco Prov.] Villa Carmen [Manu], 3 Mar 1964, C. Vargas 15199 (WAG1290507); Huánuco Dept., Pachitea Prov., 300 m a.s.l., 30 Jan 1967, J. Schunke 1579 (G); Ayacucho Region, La Mar prov., between Tambo San Miguel & Ayna, 18 Aug 1968, T.R. Dudley 11821 (F1692339); Amazonas Dept., Quebrada Huampami, 14 Nov 1972, R. Kayap 64 (F1821272); San Martín Dept., towards Santa Rosa, 350–370 m a.s.l., 9 Aug 1973, J.S. Vigo 6783 (L1690989); Loreto Dept., nr Iquitos, 1977, J. Revilla 3027 (F2202869); Loreto Dept., Maynas Prov., nr Iquitos, 120 m a.s.l., 22 May 1978, A. Gentry & al. 22206 (F2028534); Amazonas Dept., nr La Poza, 9 Aug 1979, J.A. Leveau 42 (P05003752); Loreto Dept., Caserio Sapa Playa, 15 Aug 1979, M. Rimachi 4560 (K005773557); Rio Itayu, 18 July 1981, H. Murphy 29 (K005773570); Huánuco Region, Tingo María, 700–800 m a.s.l., 9 Dec 1981, T. Plowman & al. 11242 (F1894977); Ucayali Dept., Panguana, 9 Jul 1983, F. Seidenschwarz 13/1 (F1976892); Jaén Prov., Huahuaya, 870 m a.s.l., 31 Jul 1994, S. Leiva & al. 1210 (F2154290); Bagua Prov., Imaza Distr., Yamayakat community, Jan 1995, V. Hodges & J. Gorham 130 (F2239684); Cuzco Prov., San Martin, 15 Feb 1997, P. Nuñez 19016 (US03543088).

**Saint Martin**: [without exact location] 1856, J.J. Triana 19701 (BM).

**Saint Vincent and the Grenadines**: St. Vincent Island, [without date, probably 1820s] L. Guilding s.n. (K005773490).

**Suriname (selected)**: Paramaribo, Apr 1916, J.A. Samuels 98 (K005773497); Domburg, 13 December 1960, K.U. Kramer & W.H.A. Hekking 2348 (NO0052754); nr Paramaribo, 10 January 1961, K.U. Kramer & W.H.A. Hekking 2618 (BR0000027812082); Brokopondo Distr., Brownsweg, 37 m a.s.l., 19 Aug 2006, S. Ruysschaert & al. 858 (BBS0000438).

**Trinidad and Tobago**: Trinidad, [without date] B. de Schach s.n. (K005773485); Trinidad, 17 Jan 1889, ex herb. Krug & Urban 3426 (G); Maracas Falls, 30 May 1956, Simmonds 15409 (K005773489); St. Joseph Stock Farm, 8 Mar 1958, J.W. Purseglove 6133 (K005773496, L1690992).

**Venezuela**: Aragua State, Henry Pittier NP, 850 m a.s.l., 7 Nov 1973, T. Romero 302 (F1768541); Bolívar State, Salto Pará, 80 m a.s.l., 12 Jan 1977, J.A. Steyermark & al. 112887 (F1804290); Zulia [State], distr. Bolívar, 4 Feb 1980, S. Bunting 8673 (K005773556); Yaracuy State, Bruzual distr., 12 Mar 1981, A. Julián & al. 124909 (K005773554); Portuguesa State, 30 km W of Guanare, 13 Mar 1982, R. Liesner & al. 12599 (K005773555, MA391992); The Capital Distr., Cordillera de la Costa, El Ávila NP, 200–230 m a.s.l., 14 Apr 2006, W. Meier 13874 (B100527007).

##### General distribution.

Tropical America.

#### ﻿ Cyathula aethiopica

Taxon classificationPlantaeCaryophyllalesAmaranthaceae

Sukhor. sp. nov.

D1D743B3-E487-5475-957C-D1F752D21BB0

urn:lsid:ipni.org:names:77366411-1

Fig. 5

##### Holotype.

**Figure 5.**
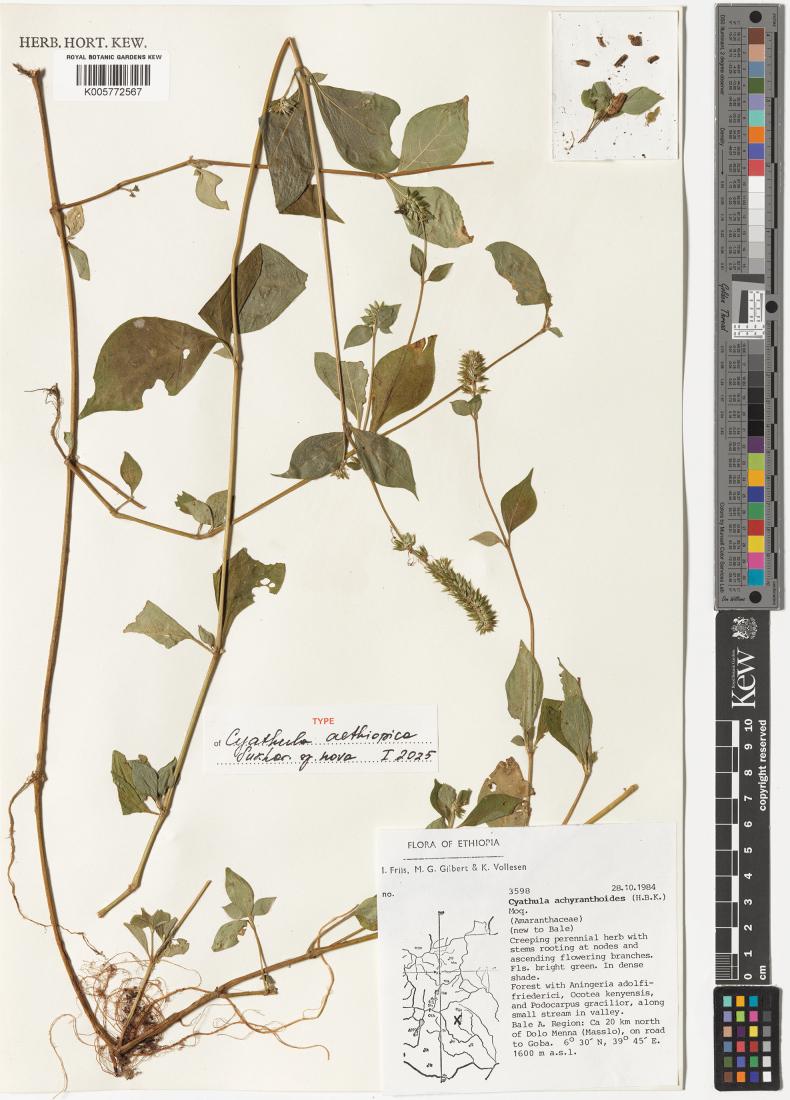
Holotype of *Cyathulaaethiopica*.

Ethiopia [Oromia Region], Bale Region [Zone], ca. 20 km north of Dolo Menna [Delo Mena] (Masslo), on road to Goba, 6°30'N, 39°45'E, forest with *Aningeriaadolfi-friederici*, *Ocoteakenyensis*, and *Podocarpusgracilior*, alt. 1600 m a.s.l., 28 October 1984, *I. Friis, M.G. Gilbert, K. Vollesen 3598* (holotype – K005772567!, isotypes – B100012020 – image seen! C – n.v.). Fig. 5.

##### Description.

***Perennial herbs*** up to 50–70 cm tall, rooting at nodes; ***stems*** upright or ascending, green or often purple-tinged, 0.6–2.0 mm in diameter, almost roundish or slightly angulate, glabrous or sparsely pubescent with simple inwardly curved or spreading hairs up to 1.0 mm long (except inflorescence axis); ***leaf pairs*** 4–6 on each branch; ***leaves*** ovate or rhombic-ovate, shortly petiolate (petioles 2.0–10.0 mm), dark green above and pale green below, cuneate, entire, 20.0–90.0 × 10.0–40.0 mm, long-acuminate, distant, uppermost leaves short-acuminate, ± close to the inflorescence; inflorescence with two paracladia forming condensed-thyrsoid synflorescence; ***main florescence*** dense or ± interrupted below, 20–60 mm long, its axis with horizontally spreading or crisp simple hairs up to 1.0(1.5) mm long; ***bract*** (subtending each cyme) persistent, 2.2–3.0 mm long, ovate with acuminate tip, pale green, sometimes with hyaline margin; ***cymes*** pedicellate (pedicels (0.5)1.0–2.0 mm long); each cyme symmetric, with two mucronulate or slightly hooked, equal, 2.0–3.0 mm long, indistinctly keeled first-ordered bracteoles (*br1*: Fig. 3C), with one fertile flower (***fertile part***) and ***two sterile parts*** in the axils of the *br1* on both sides of the fertile flower; ***second-ordered bracteoles*** of the sterile parts (*br2*) two, hyaline, glabrous to ± hairy in the lower portion (especially on keeled mid-rib), 4.0–5.0 mm long, both uncinate, with two minor (1.0–1.5 mm) hooks (third-ordered bracteoles, *br3*) in their axils; each ***sterile flower*** typically consists of five hooked perianth segments (two or three larger hooks 3.0–3.5 mm long and the others of shorter length); all larger hooks (*br2*) ± equal to perianth of fertile flower; ***perianth segments*** of fertile flower 5, 4.5–5.5 mm long, two outer segments larger than three inner segments, glabrous or shortly pubescent in their lower portion, with prominent mid-rib and two indistinct lateral veins, strongly hooked at the top or sometimes only mucronulate (both types can be present on a plant); thus, each cyme has at least 12 unequal uncinae; ***pseudostaminodes*** white, 0.5–0.7(1.0) mm long; ***anthers*** 0.4–0.5 mm long; ***style*** (with capitate stigma) 1.0–1.1 mm long; fruit (without style) 2.3–2.8 mm long; ***seed coat*** brown, thin; ***embryo*** curved; ***radicle*** pointing upwards.

##### Note.

The species is variable in having mucronate or uncinate bracteoles 1 (*br1*) and perianth segments on one individual (heteroanthocarpous plant).

##### Remark.

The ITS tree (fig. 3 in Di Vincenzo et al. (2025)) does not reveal paraphyly in *C.achyranthoides*, yet the two accessions identified under this name represent distinct species: AC1387, now recognised as *C.aethiopica* Sukhor. and AC1388, a genuine *C.achyranthoides* specimen from Venezuela.

##### Distribution

**Figure 6.**
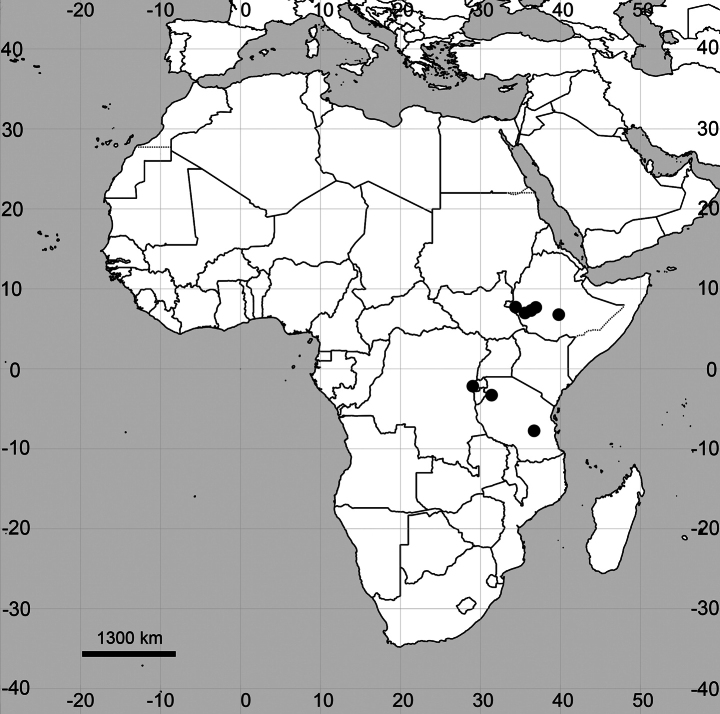
Distribution map of *Cyathulaaethiopica*.

**(Fig. 6). DR Congo**: [South Kivu Prov.] Idjwi Island, ~ 1800 m a.s.l., May 1929, H. Humbert 8408 (BR0000013780456, P05028684). Note. Perianth mucronulate, but not uncinate.

**Ethiopia**: [South West Ethiopia Peoples’ Region, Keffa Zone] Bonga, about 5 km south of Roman Catholic Mission, dense montane forest, alt. ca. 1800 m a.s.l., 21 Dec 1965, W.J.J.O. de Wilde & B.E.E. de Wilde-Duyfies 9361 (BR0000013779146, WAG0186036); [Oromia Region, Jimma Zone] ca. 57 km from Jimma on the Sheki-Gogeb River track, 7°15'N, 36°55'E, alt. 1750 m a.s.l., *Olea*–*Polyscias* forest, 8 Dec 1972, I. Friis & al. 1686 (BR0000013779139); Bale Region, Bale Mountains NP, 8 km below Rira vill., 2300 m a.s.l., 19 Oct 1988, I. Friis & al. 5593 (BR0000013779122). Note. Almost all specimens of *C.achyranthoides* cited in Townsend (2000) belong to *C.aethiopica*.

**Tanzania**: [Kagera Region] Bukoba distr., Kaloma, 1935, H. Gillman 347 (K005772946); [Iringa Region, Kilolo Distr.] Udzungwa Mountain NP, 1500 m a.s.l., 3 Oct 2001, P.A. Luke & al. 8120 (K005772943); [Iringa Region, Kilolo Distr.] Udzungwa Mountain NP, 1150 m a.s.l., 8 Jun 2002, P.A. Luke 8774 (BR0000016151345, K005772944). Note. A specimen from Tanzania collected in 1935 is the only one cited as *C.achyranthoides* by Townsend (1985).

##### Habitat.

Dense mountain forests at elevation of 1000–2400 m a.s.l.

##### IUCN category.

The new species is known from Ethiopia, Tanzania and easternmost DR Congo. Some specimens were collected in National Parks or Protected Areas where human activities are regulated. Due to a lack of information about the abundance of *C.aethiopica* in east tropical Africa, we provisionally categorise *C.aethiopica* as a Data Deficient (DD) species.

##### Relationships.

Morphologically, *C.aethiopica* is similar to *C.brevispicata* Sukhor. sp. nova and to *C.fernando-poensis* Suess. & Friedrich due to relatively short florescences and ± glabrous, hooked and large perianths of the fertile flowers. However, *C.aethiopica* has shorter styles compared to *C.brevispicata* (~ 1.0 mm vs. 1.2–1.6 mm). From *C.fernando-poensis*, the new species differs by having more hairy and usually hooked segments of fertile flowers. See also Table 1 for comparison.

**Table 1.** The main differences between the species in the *Cyathulaachyranthoides* group.

Character / Species	*Cyathulaachyranthoides* s.str.	*Cyathulageminata*	*Cyathulafernando-poensis*	*Cyathulabrevispicata*	*Cyathulaaethiopica*
Leaf petiole, mm	< 15	< 10(15–20)	< 10	< 12	< 10
Leaf blades	Oblong, apically attenuate	Obovate, apically not attenuate and shortly acuminate	Ovate, apically long-attenuate	Ovate, apically not attenuate and shortly acuminate	Ovate or rhombic-ovate, acuminate (upper leaves attenuate)
Length of the main florescence, mm	70–150(170–200)	40–100	(15)20–60	10–25(30)	20–60
Paracladia	Present	Present	Present	Absent	Present or underdeveloped
Sterile part of the cymes	Located on one side of the fertile flower	Located on one side of the fertile flower	Located on one side or on both sides of the fertile flowers	Located on one side or both sides of the fertile flowers	Located on both sides of the fertile flowers
Length of the perianth of fertile flowers, mm	3.0–4.0	3.0–4.0	5.0–5.5	4.5–6.0	4.5–5.5
Perianth tip of fertile flowers	Straight	Straight	Straight or recurved	Recurved or hooked	Usually hooked
Pubescence of the perianth of fertile flowers	Abundant with curled hairs	Abundant with curled hairs	Absent or with scarce appressed or setose hairs	Absent or with scarce appressed or setose hairs	Absent or nearly so
Style length, mm	0.6–1.0	0.8–1.0	0.7–0.9	1.2–1.6	1.0–1.1
Fruit length (without style, mm)	1.7–2.4	1.8–2.4(2.8)	2.0–2.2 (3.0 in the specimens from Cameroon)	2.0–2.5	2.3–2.8

##### General distribution.

East Tropical Africa (Ethiopia and Tanzania, probably present in Kenya, Rwanda, South Sudan and Uganda based on Fig. 6), restricted to the mountains of the East African Rift System.

#### ﻿ Cyathula brevispicata

Taxon classificationPlantaeCaryophyllalesAmaranthaceae

Sukhor. sp. nov.

D5DE1CF8-9C1D-5144-B44C-B6560B68C925

urn:lsid:ipni.org:names:77366412-1

Fig. 7

##### Holotype.

Madagascar, Province Majunga, 9.3 km NW of Ambohitsaratelo-Bebao (NW of Tsiroanomandidy), forest, ca. 1200 m a.s.l., herb in sand along creek, 15 Jan 1985, *L.J. Dorr, L.C. Barnett, A. Rakotozafy & R. Rajemisa 3562* (holotype – K005773299!, isotypes – MO, n.v., MW, TEF). Fig. 8.

**Figure 7.**
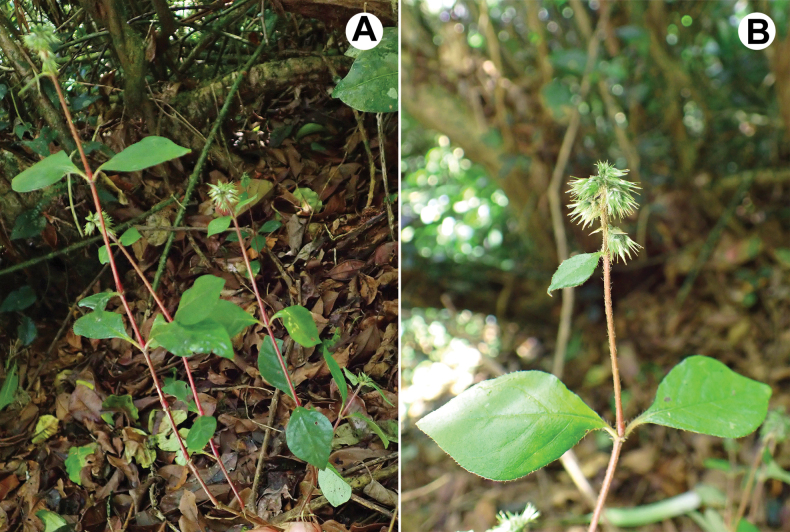
*Cyathulabrevispicata*. A. General habit; B. Inflorescence (Madagascar, Vohemar Prefecture, forêt de Sorata). Photographer: Alessandra Havinga.

**Figure 8.**
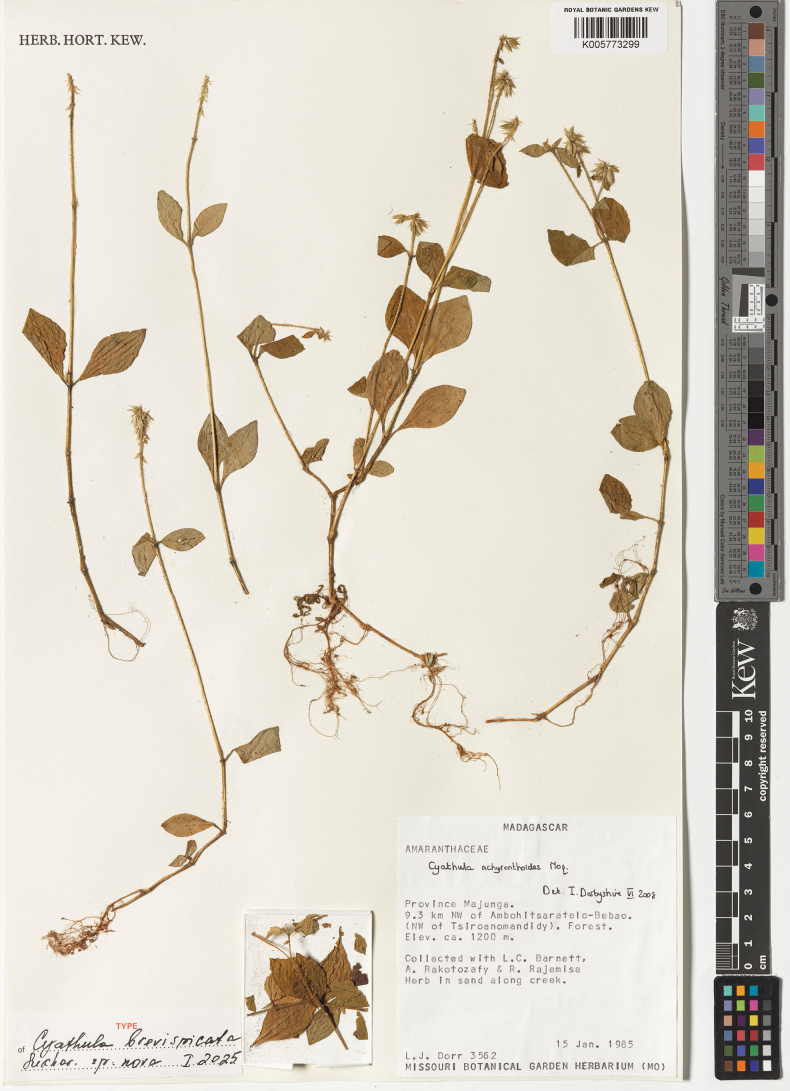
Holotype of *Cyathulabrevispicata*.

##### Description.

***Perennial herbs*** up to 30 cm high, rooting at nodes; ***stems*** thin, 0.6–1.5 mm in diameter, almost roundish or slightly angulate, sparsely pubescent with simple inwardly curved or spreading hairs up to 1.0 mm long; ***leaf pairs*** 3–6 on the stem; ***leaves*** ovate or rhombic-ovate, shortly petiolate (petioles 2.0–12.0 mm), green on both sides or brighter abaxially, cuneate, entire, 15–40 × 10–25 mm, uppermost leaves much shorter, distant from the inflorescence, hairy mostly along the veins abaxially and petioles (hairs appressed or curved, up to 1.5 mm long); ***inflorescence*** compact and dense, up to 25(30) mm long, but usually 10–15 mm long, its axis with horizontally spreading or crisped simple hairs up to 1.0(1.5) mm long, paracladia not detected; ***bract*** (subtending each cyme) persisting on the axis, 2.5–4.0 mm long, ovate with acuminate tip, hyaline; ***cymes*** (Fig. 2B) shortly pedicellate (pedicels 1.0–1.5 mm long), symmetric, consisting of one fertile flower (***fertile part***) with two mucronulate or slightly hooked, equal, indistinctly keeled first-ordered bracteoles (*br1*: Fig. 3C) 3.5–4.5 mm long, acuminate or hooked with ***two sterile parts*** in their axils on both sides of the fertile flower; two ***bracteoles*** of each sterile part (second-ordered bracteoles, or *br2*) hyaline, glabrous to ± hairy in the lower portion (especially on keeled mid-rib), 4.0–5.0 mm long, uncinate, with two axillary minor hooks (*br3*); each sterile flower (Fig. 2C) consists of five hooked perianth segments (two or three larger hooks ~ 4.0 mm long and shorter other hooks); all larger hooks (*br2*) ± equal to perianth length of fertile flower; ***perianth segments*** of fertile flower 5, 4.5–6.0 mm long, two outer segments larger than three inner segments, glabrous or pubescent in their lower portion or rarely throughout, with prominent mid-rib and two indistinct lateral veins, strongly hooked at the top; thus, each cyme has at least 12 unequal uncinae; ***pseudostaminodes*** white, 0.5–0.7 mm long; ***anthers*** 0.4–0.5 mm long; ***style*** (with stigma) 1.2–1.6 mm long; fruit (without style) 2.0–2.5 mm long; ***seed coat*** brown, thin; ***embryo*** curved; ***radicle*** pointing upwards.

##### Distribution

**Figure 9.**
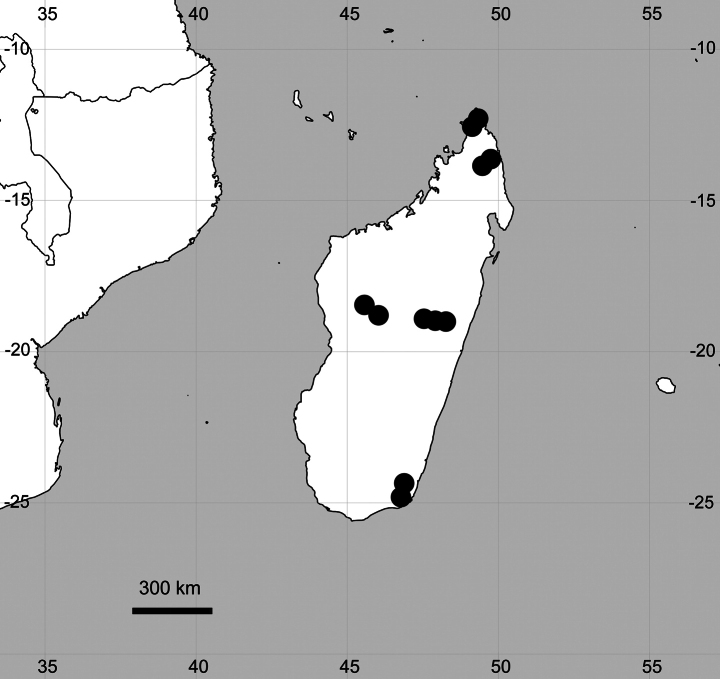
Distribution map of *Cyathulabrevispicata*.

**(Fig. 9). Madagascar**: [Analamanga Region] nr Mandraka, Aug 1906, Ch. D’Alleizette 995 (P04987035); Analamahitsy [near Antananarivo], 900 m a.s.l., Aug 1907, H. Perrier de la Bathie 8661 (P04987026); [Alaotra-Mangora Region] Analamazaotra Forest, 900 m a.s.l., 30 Oct 1912, R. Viguier & H. Humbert 1082 (P04987028, P04987039, P04987040); [Diana Region] Montagne d’Ambre, Sep 1926, H. Perrier de la Bathie 17714 (P04987024); [Anosy Region] Massif de Beampingaratra (Sud-Est), du col de Bevava au sommet de Bekoho, 1100–1500 m a.s.l., 6–7 Nov 1928, H. Humbert 6425 (K005773300); [Alaotra-Mangoro Region] Moramanga, 9 Feb 1930, R. Decary 7013 (BR0000013779177, P04987041); [Anosy Region] nr Eminiminy, 1000–1200 m a.s.l., Feb 1934, H. Humbert 14002 (P04987032, P049877033, P04987034); [Diana Region] Montagne d’Ambre, Jul 1953, J.M. Bosser 5925 (P04987029); [Diana Region] nr Diego-Suarez [Antsiranana] City, 25 Nov 1970, M. Keraudren-Aymonin & G.G. Aymonin 25582 (P04987037); [Bongolava Region] Majunga Prov., 9.3 km NW of Ambohitsaratelo-Bebao, ~ 1200 m a.s.l., 15 Jan 1985, L.J. Dorr & al. 3562 (P04987036); Diego Suarez / Antsiranana Prov., Vohemar Pref., Amboriala, forêt de Sorata, 17 Jan 2022, A.M. Havinga & Iharivolana 178 (G00409638).

##### Habitat.

Mountain forests at elevations of 900–1500 m a.s.l.

##### IUCN category.

The new species is known from twelve collections made in different parts of Madagascar. Many of them lack precise coordinates, making it impossible to determine whether they are located within protected areas. Due to the lack of information about the abundance of *C.brevispicata* in Madagascar, we provisionally categorise it as a Data Deficient (DD) species.

##### Relationships.

Morphologically, the species is very close to *C.aethiopica* Sukhor. sp. nova, but differs by shorter inflorescence without paracladia and longer style.

##### General distribution.

Endemic to Madagascar.

#### ﻿ Cyathula fernando-poensis

Taxon classificationPlantaeCaryophyllalesAmaranthaceae

 Suess. & Friedrich, Mitt. Bot. Staatssamml. München 1(6): 188 (1953).

E047AC9E-523E-5D68-9AAC-B7177195D957

##### Holotype.

Equatorial Guinea, Fernando Po [Bioko] Island, El Pico, 7000 ft [2133 m], in forest, 10 Dec 1951, A.S. Boughey 123 (K000243618).

##### Note.

The type designation for this species name is ambiguous. Suessenguth (1953) stated that the type was collected at an elevation of 7000 ft [2133 m]. and cited two collection numbers, *Boughey 123* and *124*, but only the former agrees with the stated elevation and is labelled as type at K. We therefore assume that the added citation of the collection number 124 was a technical error that does not affect the type designation.

##### Description.

***Perennial herbs*** up to 60 cm high, rooting at nodes; ***leaves*** short-petiolate (petioles up to 10 mm long), rhombic, obovate or elliptic, 30–60 × 20–30 mm, hairy, upper leaves ± distant from the inflorescence, hairs on leaves articulated, 0.5–1.1 mm long; ***bracts*** persisting on the florescence axis, ovate, 2.0–3.0 mm long, short to long acuminate, glabrous or ± setose; ***main florescence*** (15)20–60 cm long, dense or slightly interrupted basally, with shorter paracladia; ***cymes*** (Fig. 2D) shortly pedicellate (pedicels 0.5–1.0 mm long), symmetric, with two mucronulate or slightly hooked, equal, indistinctly keeled first-ordered bracteoles (*br1*: Fig. 3C) 2.0–3.0 mm long and fertile flower in between (***fertile part***) and mostly ***two sterile parts*** located in the axils of *br1* on both sides of the fertile flower, sometimes only one sterile part is present in a cyme and, thus, the cyme is asymmetric; paired second-ordered ***bracteoles*** (*br2*) of the sterile parts hyaline, glabrous, short (2.5–3.0 mm), not exceeding the fertile flower, uncinate, with minor two hooks (*br3*) in their axils, sterile part consisting of a sterile flower with five apically recurved or straight segments: two outer segments of 4.0 mm long and three inner smaller (3.0 mm long) segments; all larger hooks (*br2*) ± equal to length of perianth of fertile flower; ***perianth segments*** of fertile flower 5, 5.5–5.5 mm long, glabrous or ± pubescent only basally, each segment with prominent mid-rib and two indistinct lateral veins; ***pseudostaminodes*** 1.0–1.2 mm long; ***anthers*** 0.4–0.5 mm long; ***style*** (with capitate stigma) 0.7–0.9 mm long; ***fruit*** (without style) 2.0–2.2(3.0) mm long; ***seed coat*** brown, thin; ***radicle*** pointing upwards.

##### Note.

Suessenguth & Friedrich in Suessenguth (1953) described the species, based on three examined specimens. Having examined the subsequent collections from Cameroon, we can improve the description of *C.fernando-poensis*. For example, Suessenguth & Friedrich in Suessenguth (1953) indicated that there are only two hooks in a sterile flower. However, the number of hooks in a cyme can vary from 2 to 8. Two hooks are present if there is only one sterile part in a cyme and no other additional hooks are present in the bract axil.

##### Habitat.

(Sub)mountain forests at elevations of 1300–2800 m a.s.l.

##### IUCN category.

In Cameroon, *C.fernando-poensis* was first assessed as Vulnerable (Darbyshire in Cheek et al. (2004); Onana and Cheek 2011). Nevertheless, Onana and Cheek (2011) indicated that the forests of the South-West Region of Cameroon and Bioko over 1000 m a.s.l. were, at that time, under minimal anthropogenic pressure. Later, the conservation status of *C.fernando-poensis* in Cameroon was changed to Endangered (Cheek 2014), because the populations are under great pressure of clearance for agriculture and this threat occurs across the whole range and even inside areas that are designated as “protected”. However, at that time, the existence of the population in North-West Region (where it has been most frequently collected) was unknown due to misidentification. There are no data on the abundance of the species in Bioko Island (Velayos et al. 2013).

##### Distribution

**Figure 10.**
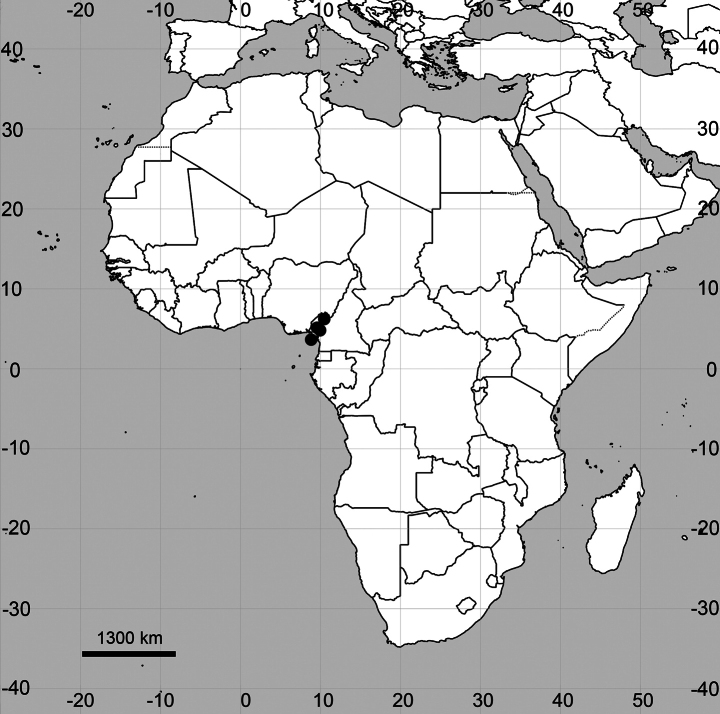
Distribution map of *Cyathulafernando-poensis*.

**(Fig. 10). Equatorial Guinea**: Fernando Po [Bioko] Island, El Pico, 6600 ft [2012 m], in forest, darker variety of [the specimen number] 123, 10 Dec 1951, A.S. Boughey 135 (K000518886).

**Cameroon**: South-West Prov., Mount Kupe, Max’s trail leading from Nyasoso to summit of Mt. Kupe, 4°48'N, 9°41'E, 1550 m a.s.l., closed canopy submontane forest on fertile micro-aggregated humic cambisol, 22 Nov 1994, P. Lane 242 (K000086531, SCA,YA); North-West Region, Bui Dept., Elak, 2200 m a.s.l., montane forest, 9 Jun 1996, S. Cable & al. 2964 (K, MW, YA); North-West Region, Bui Dept., Elak, Mt. Oku, 2500 m a.s.l., montane forest, 9 Jun 1996, L. Zapfack 800 (BR, K001900912, MW, SCA,YA); North-West Region, Bui Dept., Elak, 2250 m a.s.l., montane forest, 9 Jun 1996, S. Cable 2975 (K001900914, MW, YA); North-West Region, Bui Dept., Oku-Elak, 6.1349°N, 10.3112°E, 2200 m a.s.l., lower parts of transect KA, 28 Oct 1996, M. Cheek 8491 (K001900858, MW, YA); North-West Region, Bui Dept., Elak-Oku, 6.15°N, 10.26°E, 2500 m a.s.l., K.A path, 29 Oct 1996, J.M. Onana 460 (K001900862, MW, WAG); North-West Region, Bui Dept., Elak, 2800 m a.s.l., 6.1349°N, 10.3112°E, grassland, 30 Oct 1996, M. Cheek & al. 8519 (K001900859, MW); North-West Region, Bui Dept., Oku-Elak, 2600 m a.s.l., Transect KA, mountain slope, open forest, 31 Oct 1996, M. Buzgo, 689 (BR, K001900860, MW, YA); South-West Prov., Kupe-Muanenguba Division, Kodmin, 5°00'00"N, 9°41'11"E, alt. 1330 m a.s.l., montane / submontane evergreen forest, road to Mwanzum and Nyale, 8 Dec 1999, M. Cheek & al. 10269 (K000051086, K000051088).

##### General distribution.

For a long time, this species was considered endemic to Bioko Island, Equatorial Guinea (Exell 1973; Brenan 1978). In the 1990s, *C.fernando-poensis* was also discovered at Mt Kupe and the Bakossi Mts of South-West Region in Cameroon (Cheek et al. 2004). However, the records from North-West Region were until now misidentified as *Achyranthesaspera* L. (Cheek et al. 2000).

##### Remark.

*Cyathulageminata* and *C.fernando-poensis* are both present in Bioko Island and Cameroon. They differ in morphology and altitudinal preferences. *Cyathulageminata* is a typical component of drier tropical rainforests, up to an upper altitudinal limit of 1200 m a.s.l., while *C.fernando-poensis* is found in the mountain rainforests. Further, in Cameroon, *C.geminata* is not recorded from the high rainfall forests of SW Region (e.g. Cable and Cheek (1998)), but only in the lower rainfall, often semi-deciduous forest areas of Central, West and South Regions (e.g. Cheek et al. 2011), albeit misidentified as a variant of *C.prostrata* (L.) Blume.

#### ﻿ Cyathula geminata

Taxon classificationPlantaeCaryophyllalesAmaranthaceae

(Schumach.) Moq. in DC., Prodr. 13(2): 330 (1849).

1D9D5B31-A4B4-5C4E-BE48-8F43D4C9B43F

≡ Achyranthesgeminata Schumach., Beskr. Guin. Pl.: 138 (1827). Described from Ghana (Akuapem-Akropong, Eastern Region).

##### Note.

Schumacher (1827) noted that the original material of the species collected in present-day Ghana and described — but not named — by P. Thonning had largely been lost during the bombardment of Copenhagen in 1807, amidst the Napoleonic Wars. This loss had already occurred by the time Schumacher validly published numerous new species names in that work. Later, Hepper (1976) confirmed that no material of *Achyranthesgeminata* Schumach. is preserved at C, supporting the likelihood that it was destroyed during the war. Although the taxonomic identity of *A.geminata* is beyond doubt, thanks to the detailed morphological description provided in its protologue, we refrain from designating a neotype due to the current scarcity of herbarium collections from Ghana.

The name *C.geminata* was rarely discussed in early literature. Hooker (1849) and Baker and Clarke (1909) accepted it, but did not mention the name *C.achyranthoides* and rather believed that *C.geminata* is hardly separable from *C.prostrata*. Cavaco (1954) placed *C.geminata* in the synonymy of *C.prostrata*, but later merged it with *C.achyranthoides* (Cavaco 1962: 85) and also cited it together with *C.prostrata* and *C.achyranthoides* in the list of Amaranthaceae occurring in Fernando Po (Bioko) Island, Equatorial Guinea, without any explanation (Cavaco 1962: 221). Subsequent authors (e.g. Gosline et al. (2023)) have consistently referred to the taxon as *Cyathulaachyranthoides*, maintaining it as distinct from *C.prostrata*.

##### Description.

***Perennial herbs*** up to 1 m high rooting at nodes; ***leaf pairs*** 3–6 on each branch; ***leaves*** obovate or broadly ovate, shortly petiolate (petioles up to 10(15–20) mm), apically not attenuate and shortly acuminate, 40–130 × 20–50 mm, glabrous or sparsely pubescent (mostly abaxially on the veins) with appressed hairs up to 1.0 mm long; ***bract*** (persistent on the inflorescence axis) 1.5–2.0 mm long, triangular or oblong; synflorescences dense, ***main florescence*** 40–100 mm long; paracladia present; cymes (Fig. 2E) asymmetric, bracteoles of the first order (*br1*: Fig. 3A, B) two, ~ 2.5 mm long, narrowly oblong, mucronate, with central fertile flower in between, ***sterile part*** pedicellate (pedicel 0.5–0.8 mm long), with central sterile flower having a narrowly cylindrical perianth up to 2.0(2.5) mm long, its segments lanceolate, acuminate, non-hooked or only recurved; second-ordered bracteoles (*br2*) two, uncinate, 2.5–3.0 mm long; two rudimentary flowers located in the axils of second-ordered bracteoles are accompanied by paired uncinate third-ordered bracteoles (*br3*) shorter than *br2*; thus, each cyme has at least six unequal uncinae; ***perianth of fertile flower*** with five green segments 3.0–4.0 mm long, pubescent, each segment three-nerved; ***pseudostaminodes*** 0.3–0.4 mm long, entire; ***anthers*** ~ 0.3 mm long; ***style*** (with capitate stigma) 0.8–1.0 mm long; ***fruit*** (without style) 1.8–2.4(2.8) mm long; ***seed coat*** brown; ***radicle*** pointed upwards.

##### Habitat.

Tropical lowland and submontane forests up to 1200 m a.s.l., semi-shady disturbed areas.

##### IUCN category.

Frequently found in ruderal sites (e.g. Hauman (1951); Chatelain et al. (2024), both as *C.achyranthoides*) and, therefore, is assigned the least-concern (LC) category.

##### Distribution

**(Fig. 11).**

**Figure 11.**
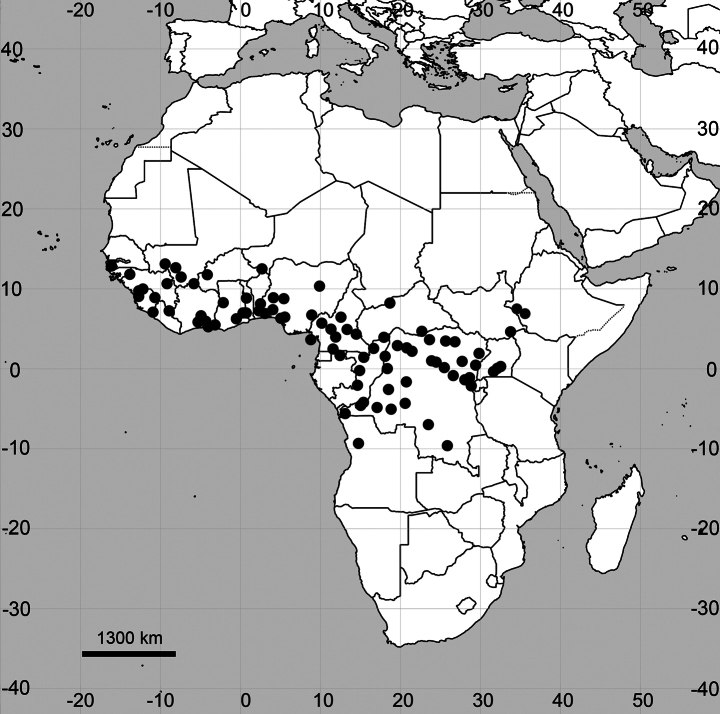
Distribution map of *Cyathulageminata*.

**Angola**: [Cuanza Norte Prov.] Cazengo, May 1912, J. Gossweiler 5705 (COI00068665).

**Benin**: [without exact location and date] M. Poisson s.n. (K005772342); [Plateau Dept.] Pobé City, 2 Sep 1964, E.J. Adjanohoun 120 (P05029074); Zou Dept., Cové, 12 Dec 1998, N. Sokpon 464 (BR0000013779054); Zou Dept., Savè, 15 Dec 1999, V. Adjakidjè & al. 3178 (WAG0204798).

**Burkina Faso**: Houet [Prov.], 80 km NE of Bobo[-Dioulasso], 23 Jan 2005, L. Sanou & M. van Slageren 1662 (K005772326).

**Cameroon (selected)**: [East Region] 30 km SSE of Batouri, 7 Apr 1962, R. Letouzey 4677 (P05028675); [Centre Region] nr Ngoro vill., 30 Mar 1963, J. Raynal & A. Raynal 10594 (P05028673); [Adamawa Region] Tibati, Sep 1963, R. Letouzey 5846 (P05028679); [East Region] Njangené, S of Ebaka, 27 Dec 1965, A.J.M. Leeuwenberg 7371 (K005772496, P05028683); [West Region] Bamboutos Mt., 10 Sep 1967, A. Meurillon 603 (K005772495); [South Region] Nkolowom, 10 Mar 1970, R. Letouzey 110129 (K005772494); [Central Region] Ndanan, M’Fou Park, 9 Mar 2004, M. Cheek & al. 11577 (K000339697).

**Central African Republic**: Ouaka Pref., Oubangui plateau, 20 Mar 1925, R.P.Ch. Tisserant 1700 (P05028702); [Lobaye Pref.] Mbaïki, 3 Dec 1936, R.P.Ch. Tisserant 3454 (P05028700).

**Chad**: Reported by César and Chatelain (2019); probably a collection “[Nana-Grébizi Pref.] Chari Territory, Between Fort Crampel [Kaga-Bandoro, Central African Republic] town & La Nana [N’Gueli, Chad Rep.], Dec 1903, A. Chevalier 10689” (P05028703) belongs hereto.

**Congo Rep.**: Nkéni-Alima Dept., Gamboma to Etoro, 7 Jun 1961, B. Descoings 7108 (P05028696); [Cuvette-Ouest Dept.] Ewo to Palabaka, 3 Aug 1961, B. Descoings 8738 (P05028685); [Likouala Dept.] nr Impfondo, 20 Jan 1966, A. Bouquet 2008 (P05028697); M’Bamou Island, 25 May 1967, P. Sita 1701 (P05028692); Sangha Dept., Nouabalé-Ndoki NP, 379 m a.s.l., 20 Dec 2011, N. Ebika 688 (E00737319, WAG1971816); Sangha Dept., Bon Coin vill., 1 Feb 2013, N. Ebika 974 (E00757912, WAG1576459).

**Figure 12.**
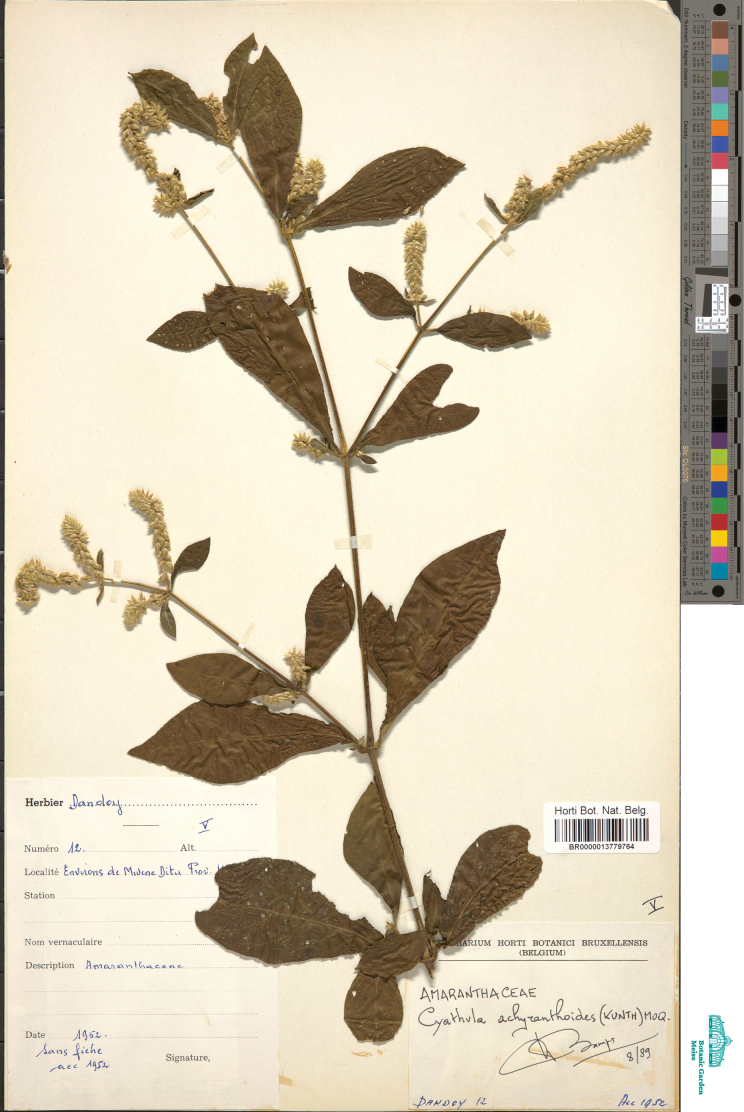
A representative specimen of *Cyathulageminata*.

**DR Congo (selected)**: [Tshopo Prov.] Stanleyville [Kisangani], 12 Jan 1882, P.T.L. Putman 93 (K005772497); [Bas-Uélé Prov.] Dili, [without date, probably early 20^th^ century] K. Jespersen 25 (BR0000013780074); [Kasai Prov.] Port Francqui [Ilebo], [without date, probably early 20^th^ century] H. Vanderyst 24370 (BR0000013779689); [Sud-Ubangi Prov.] Bokumu, [without date, probably early 20^th^ century] H. Vanderyst 24527 (BR0000013779733); [Équateur Prov.] Eala, Nov 1923, V. Goossens 4452 (BR0000013779993); [Mongala Prov.] Lisala, Mar 1924, V. Goossens 4133 (BR0000013780029); [North Kivu Prov.] E of Beni, 1929, H. Humbert 8755 (BR0000013780463, P05028688); [Kongo Central Prov.] Kanga, 10 Oct 1930, H. Vanderyst 26162 (BR0000013779245); [Bas-Uélé Prov.] Bambesa, 19 Mar 1935, Steyaert 25 (BR0000013780364); [Haut Uele prov.] Nganzi, 30 Oct 1938, J. Louis 12186 (BR0000006607135, K005772498); [Tshopo Prov.] Yangambi, 17 Jun 1938, J. Louis 9817 (BR0000013780227, K005772501); [Sud-Ubangi Prov.] Budjala Territory, Wadju, 3 Feb 1938, V.A. Brimeyer s.n. (BR0000013779856); [Kwilu Prov.] Kikwit, 4 Jun 1946, M. Renier 89 (BR0000013779610); [Lomami Prov.] nr Mwene-Ditu, 1952, Dandoy 12 (BR0000013779764) – Fig. 12; [Haut-Lomami Prov.] Mukulakulu, 25 Mar 1953, De Troyer 68 (BR0000013779771); [Mai-Ndombe Prov.] Irongo Terr., Panza, 25 Jun 1953, G. Gilbert 14270 (BR0000013779917, WAG0186660); [South Kivu Prov.] Kalehe Territory, 3 Jan 1958, R. Gutzwiller 2515 (WAG0186661); [North Kivu Prov.] Lungoma, 1050 m a.s.l., 11 Jun 1958, R. Gutzwiller 3084 (BR0000013779986, WAG0186663); [Tshuapa Prov.] Monkoto, 9 Aug 1958, C. Evrard 4568 (BR0000013779900); [Bas-Congo Prov.] Luki vill., 17 Aug 1959, P. Compere 103 (BR0000013779207); Kwango Prov., nr Kenge, 14 May 1964, L. Pauwels 4459 (BR0000013779597); [Tshopo Prov.] 12 km E of Waine Rukula, 3 Jan 1973, S. Lisowski 15626 (BR0000013780135); [Bas-Uélé Prov.] 130 km NW of Likati, Bongbeto, 3 Jan 1978, S. Lisowski 46673 (BR0000013780173); [Tshopo Prov.] 15 km E of Kisangani, 30 Nov 1981, S. Lisowski 66797 (BR0000013780180); [Maniema Prov.] nr Lubutu, 20 Feb 1982, L. Pauwels 6588 (WAG0186662); [North Kivu Prov.] Nyamakombola, 15 Oct 1989, Terashima 8 (BR0000013780371); Mambasa Territory, Ituri Forest, 13 Apr 2001, F. Bujo Degho 632 (K005772506); [Tshopo Prov.] Liambe, 370 m a.s.l., 23 May 2010, Boyekoli Ebale Congo Expedition 754 (BR0000005706723).

**Equatorial Guinea**: Fernando Po [Bioko Island], 1843, Vogel [ex herb. Hooker] 253 (K005772356, K005772357); [location unknown] 450 m a.s.l., 18 Mar 1908, G. Tessmann 273 (K005772350).

**Ethiopia**: Southwest Ethiopia Region, Keffa Zone, S of Mizan Teferi, 1050 m a.s.l., 1 Dec 1984, I. Friis & al. 3914 (K005772572); [Gambela Region] 15 km E of Pugnido, 600 m a.s.l., 23 Nov 1995, I. Friis & al. 7289 (K005772568).

**Gabon**: Woleu-Ntem Prov., Limbali Forest, 20 Dec 1990, J.J. Dibata D57 (WAG0126503).

**Ghana**: Gibi, 16 Dec 1952, J.K. Morton 8112 (K005772328); [Eastern Region] nr Asiakwa, 19 Dec 1952, J.K. Morton 8209 (K005772329); Banda area, Bui, 20 Dec 1953, J.K. Morton 25075 (K005772327, WAG0186027); Volta Region, Kpando, 215 m a.s.l., 23 Dec 1972, J.F. Veldkamp 6092 (L1690977).

**Guinea**: Milo River, 2 Dec 1966, S. Lisowski 81483 (BR0000013779061); Forécariah Pref., nr Dalonia, 900 m a.s.l., 19 Jan 2013, M. Cheek & al. 16970 (K000334927).

**Guinea-Bisau**: Gabú Region, 5 km ESE of Lugajole, 9 Dec 2017, E.L.A.N. Simons & al. 2239 (WAG1977274).

**Ivory Coast**: [Abidjan Autonomous Distr.] Bingerville, Dec 1906, A. Chevalier 16025 (P05029080); [Abijan Autonomous Distr.] Anguédédou Forest, 1948, G. Mangenot & *L. Aké Assi 3933* (P05029082, UCJ000657); Toumodi, 4 Nov 1950, G. Roberty 12588 (G); [Agnéby-Tiassa Region] Anno vill., 15 Dec 1955, L. Aké Assi 3512 (UCJ000658); Orumbo Boka Forest, 10 Dec 1957, E. Adjanohoun 2049 (UCJ000659); Lamto, 1973, L. Aké Assi s.n. (UCJ000660); Abijan, Banco Forest Reserve, 23 Mar 1976, J. de Koning 6720 (BR0000016151369, WAG0098800); Bouna Pref., Comoé Sud, 4 Nov 1987, P. Poilecot 39755 (G); Bouna, Divo Dept., 4 km W of Divo city, 2 Dec 1992, C. Chatelain & H.G. Téhé 40288 (G).

**Liberia**: Grand Cape Mount Co., Ganna Tanyehun, 21 Dec 1947, J.T. Baldwin 10777 (K005772337); Ganta Town, 16 Dec 1951, A.J.D. Baker 1126 (K005772336).

**Mali**: [Kayes Region] Kita, 27 Dec 1947, G. Roberty 10246 (G); Bamako, 29 Nov 1969, N. Diarra 387 (P05029066); [Sikasso Region] Kadiolo, 30 Dec 1969, N. Diarra 452 (P05029067); Sikasso Region, Bougouni, 325 m a.s.l., 1 Dec 2010, S. Sanogo & al. 724 (K005772325).

**Niger**: W National Park, 8 Dec 1978, M. Saadou & M. Garba 1099 (P05029084, WAG0033827).

**Nigeria**: [Oyo State] Garuba, Sep 1890, A. Moloney 11 (K005772315); [Ogun State] Akilla, 23 Feb 1931, W.D. MacGregor 499 (K005772345); [Oyo State] nr Ibadan, 600 ft [183 m], Nov 1936, R.J. Newberry & A.E. Etim 168 (K005772313); [Edo State] Okomu Forest Reserve, 20 Jan 1948, J.P.M. Brenan & P.W. Richards 8850 & 8851 (K005772346, P05029068); [Bauchi State] Bauchi city, 4 Dec 1971, M.G. Latilo 64739 (K005772341); [Cross River State] Ogoja, nr Yahe vill., 21 Feb 1973, Latilo & Oguntayo 67625 (K005772351); [Ogun State] Ilaro Forest Reserve, 12 Dec 1982, J. Lowe 4358 (K005772349).

**Senegal**: [Ziguinchor Region] Brin vill., 27 Dec 1976, C. Vanden Berghen 1638 (BR0000013779023, WAG0105288); [Ziguinchor Region] Badiouré, 29 Nov 1983, C. Vanden Berghen 4066 (BR0000013779016).

**Sierra Leone**: Ndoke, 1914, N.W. Thomas 4537 (K005772340); [Northern Prov.] Gbinti (Dibia), 11 Jan 1953, F.C. Deighton 5900 (K005772339); Northern Prov., Dumbala to Wania, 5 Jan 1964, Morton & Gledhill 519 (K005772333); Tingi Mts., 19 Dec 1965, Morton & Gledhill 3208 (K005772330).

**South Sudan**: [Eastern Equatoria State] Talanga, 110 m a.s.l., 7 Dec 1980, I. Friis & K. Vollesen 755 (BR0000016151352, K005772565).

**Togo**: [Plateaux Region] Kloto, 1977, J.F. Brunel 4130 (TOGO01696); Nuivé, Dec 1978, J.F. Brunel 6219 (TOGO01694); Boulohou, Nov 1984, J.F. Brunel 9107 (TOGO01693).

**Uganda**: [Central Region] Buddo, 3800 ft [1158 m], Aug 1937, anonymous 1814 (K005772942); Buganda prov., Kyagwe, 3750 ft, 7 Dec 1949, H.C. Dawkins 469 (K005772940); [Central Region] 2 km E of Kayugi, 1150 m a.s.l., 22 May 1972, K.A. Lye 6833 (K005772938); [Central Region] Mawokota County, Bunjako Island, 1143 m a.s.l., 13 Mar 2011, S. Santini 457 (FT0004990).

##### General distribution.

Only known in Tropical Africa. *Cyathulageminata* is not reported for the flora of tropics of southern Africa (Phiri 2005; Odorico et al. 2022). It is also not reported for Rwanda (Townsend 1985; present paper), although its presence is possible due to records in the neighbouring regions of Uganda and DR Congo.

## ﻿Discussion

### ﻿Morphological and chorological relationships between the species of Cyanthula achyranthoides group

Five species within the *C.achyranthoides* group should be recognised: four species in Africa and one in tropical America. Based on morphology, these species can be divided into two groups: (1) a group with asymmetric cymes and smaller acuminate perianths of the fertile flowers (*C.achyranthoides*, *C.geminata*) and (2) a group with usually symmetric cymes and longer and often uncinate perianths of the fertile flowers (*C.aethiopica*, *C.brevispicata*, *C.fernando-poensis*). The most striking differences between these two groups are the length of the main florescence, the number of sterile parts in a cyme and perianth characters (length, pubescence and presence of uncinae) of the fertile flowers (Table 1). Notably, the species within each group are very similar in morphology despite their geographical isolation.

The first group consists of the lowland species: the American *C.achyranthoides* s.str. and the African *C.geminata*, which differ in leaf shape. It should be noted that some *C.geminata* specimens from Benin (BR0000013779054, K005772342) and Ghana (K005772327) have oblong upper leaves very similar to those of *C.achyranthoides* s.str. As both specimens only include the upper parts of the plant, without lower leaf pairs, the observed similarity in upper leaf shape could either reflect variation within *C.geminata* or point to a possible first record of the American *C.achyranthoides* in Africa. Further study is needed to evaluate both possibilities.

We cannot confirm the alien status of *C.achyranthoides* in America as proposed earlier (Borsch 2001; Funk et al. 2007), although the species may be allochthonous in some parts of the continent. *Cyathulageminata* is distributed in tropical West-Central Africa, but is absent eastwards of the East African Rift. All literature records from Tanzania and almost all from Ethiopia (Townsend 1985, 2000) belong to *C.aethiopica*, a new mountain species. Two other mountain species, *C.fernando-poensis* and *C.brevispicata*, are restricted to Bioko Island/Western Cameroon and Madagascar, respectively.

This ‘mountain’ group, which includes *C.aethiopica*, *C.brevispicata* and *C.fernando-poensis*, is quite uniform morphologically despite the large geographical disjunction between the species. Nevertheless, based on all specimens seen, *C.brevispicata* has the shortest inflorescences without paracladia and the longest styles. *Cyathulafernando-poensis*, compared with both *C.brevispicata* and *C.aethiopica*, seems to have much shorter bracteoles of the second order (*br2*), but this observation needs further examination on specimens collected in the fruiting stage when the sterile parts of the cymes may slightly elongate to promote epizoochorous dispersal.

### ﻿Inflorescence structure

Partial florescences are key morphological traits for distinguishing the species under study. While the number of fertile flowers per cyme (Hutchinson and Dalziel 1927; Hauman 1951; Duke 1961; Cavaco 1962) is one aspect of their differentiation, it represents only part of the variation in their inflorescence architecture. Eliasson (1988) previously suggested that the sterile portion of the cyme may be composed of modified bracteoles. However, in the *Cyathulaachyranthoides* group and in the related *C.prostrata*, the sterile structures consist of bracteoles of various orders, sterile flowers with a well-developed perianth and rudimentary flowers lacking a perianth (Fig. 3).

The three-flowered cymes of *C.fernando-poensis*, *C.aethiopica* and *C.brevispicata* are symmetric. The central (fertile) flower is complete, with either acuminate (*C.fernando-poensis*) or hooked perianth segments (obligate in *C.brevispicata* and facultative in *C.aethiopica*). Two lateral sterile flowers are present, each equal in size to the central flower; their segments are bent inwards in *C.fernando-poensis* and hooked in *C.aethiopica* and *C.brevispicata*.

In contrast, the two-flowered cymes of *C.achyranthoides* and *C.geminata* are asymmetrical and structurally distinct from those of the three aforementioned species. The central (fertile) flower is complete, with acuminate tepals; each sterile part is positioned on one side of the fertile flower and comprises uncinate bracteoles and sterile flower with three elliptic, obtuse segments and two longer, narrower, acute segments bent inwards at the apex.

A different florescence architecture is found in *C.prostrata*, which is closely related to at least *C.achyranthoides* and *C.aethiopica* (Di Vincenzo et al. (2025); both samples cited under *C.achyranthoides*). The cymes of *C.prostrata* (Fig. 3F) are bilaterally symmetric and contain three fertile flowers: one central flower lacking sterile appendages and two lateral fertile flowers accompanied by sterile ones. All fertile flowers are morphologically similar, each with lanceolate, scarious, acuminate segments; paired bracteoles *br1* and *br2* are similar, acuminate and shorter than the perianths of the flowers. All sterile flowers are transformed into pentamerous clusters with hooked outgrowths, situated in the axils of second- and third-ordered bracteoles; bracteoles *br3* are shorter than *br1* and *br2*.

Regarding propagule dispersal, the enlarged second-ordered bracteoles (*br2*), which extend beyond other parts of the cyme, likely play a key role in anchoring propagules to animal vectors.

## ﻿Conclusions

Our morphological analysis of *C.achyranthoides* s.l. revealed greater species diversity than previously recognised. Florescence architecture proves to be a key diagnostic feature within this group. The sterile portions of the cymes are composed of bracteoles of various orders, as well as both sterile and rudimentary flowers. The principal distribution area of this group lies in tropical Africa and Madagascar. A similar re-evaluation of other *Cyathula* groups would be highly desirable.

## Supplementary Material

XML Treatment for Cyathula achyranthoides

XML Treatment for Cyathula aethiopica

XML Treatment for Cyathula brevispicata

XML Treatment for Cyathula fernando-poensis

XML Treatment for Cyathula geminata

## ﻿Additional information

### Use of AI

No use of AI was reported.

### Author ORCIDs

Alexander P. Sukhorukov https://orcid.org/0000-0003-2220-826X

Alexander N. Sennikov https://orcid.org/0000-0001-6664-7657

Martin Cheek https://orcid.org/0000-0003-4343-3124

Louis Nusbaumer https://orcid.org/0000-0001-8350-5925

Maria Kushunina https://orcid.org/0000-0003-2541-6494

Alexander C. Timonin https://orcid.org/0000-0002-6066-551X

### Data availability

All of the data that support the findings of this study are available in the main text.
